# Serum Adiponectin, Vitamin D, and Alpha-Fetoprotein in Children with Chronic Hepatitis C: Can They Predict Treatment Response?

**DOI:** 10.1155/2015/617623

**Published:** 2015-11-10

**Authors:** Mohamed Ahmed Khedr, Ahmad Mohamed Sira, Magdy Anwar Saber, Gamal Yousef Raia

**Affiliations:** ^1^Department of Pediatric Hepatology, National Liver Institute, Menofiya University, Shebin El-koom, Menofiya 32511, Egypt; ^2^Department of Clinical Pathology, National Liver Institute, Menofiya University, Shebin El-koom, Menofiya 32511, Egypt

## Abstract

*Background & Aims*. The currently available treatment for chronic hepatitis C (CHC) in children is costly and with much toxicity. So, predicting the likelihood of response before starting therapy is important. *Methods*. Serum adiponectin, vitamin D, and alpha-fetoprotein (AFP) were measured before starting pegylated-interferon/ribavirin therapy for 50 children with CHC. Another 21 healthy children were recruited as controls. *Results*. Serum adiponectin, vitamin D, and AFP were higher in the CHC group than healthy controls (*p* < 0.0001, *p* = 0.071, and *p* = 0.87, resp.). In univariate analysis, serum adiponectin was significantly higher in responders than nonresponders (*p* < 0.0001) and at a cutoff value ≥8.04 ng/mL it can predict treatment response by 77.8% sensitivity and 92.9% specificity, while both AFP and viremia were significantly lower in responders than nonresponders, *p* < 0.0001 and *p* = 0.0003, respectively, and at cutoff values ≤3.265 ng/mL and ≤235,384 IU/mL, respectively, they can predict treatment response with a sensitivity of 83.3% for both and specificity of 85.7% and 78.6%, respectively. In multivariate analysis, adiponectin was found to be the only independent predictor of treatment response (*p* = 0.044). *Conclusions*. The pretreatment serum level of adiponectin can predict the likelihood of treatment response, thus avoiding toxicities for those unlikely to respond to therapy.

## 1. Introduction

Although chronic hepatitis C (CHC) in children is usually asymptomatic, it is often persistent and is a possible cause of morbidity in later life. Moreover, progressive liver disease, including cirrhosis, has been reported during childhood [1]. Although new direct-acting antivirals (DAAs) are now the cornerstones in the treatment of HCV infection in adults, combination therapy with pegylated interferon (Peg-IFN) alpha and ribavirin (RBV) (Peg/RBV therapy) is still the current standard therapy in children [2, 3]. Although this regimen was found to be highly effective in children with genotypes 2 and 3 [4], it has limited efficacy for those with genotype 4 [5, 6], the prevalent genotype in Egypt [7]. Therefore, the ability to predict response to this costly with many side effects antiviral regimen remains an important research goal.

In spite of the extensive researches in this field, knowledge is still defective. On defining predictors of treatment response, many studies concentrated on virus genotype, gender, age, race, and fibrosis stage, among others. Nevertheless, all of these are nonmodifiable factors but only give a prediction about the likelihood of sustained virologic response (SVR). Moreover, some of them are expensive and invasive [8]. On investigating the role of* interleukin-28B* (*IL28B*) polymorphism in pediatric patients with CHC, one study suggested that* IL28B* genotype was not a strong predictor of SVR [9], while other studies have shown usefulness of* IL28B *genotype as a predictor of treatment response [10–12].

Virological responses during therapy, such as rapid virologic response (RVR) and early virologic response (EVR), are widely used for predicting end of treatment response (ETR) and SVR [13]. Nonetheless, it is obvious that predictions made before administration of therapy are more desirable than those done during treatment course.

We aimed to investigate some inexpensive, easy to perform, and noninvasive modifiable (as adiponectin and vitamin D) and nonmodifiable (as alpha-fetoprotein “AFP”) factors which may have a relation to treatment response in children with CHC. If we found a significant relation to successful treatment, we can use them not just to predict the likelihood of treatment response but also to improve the SVR rates by modulating the modifiable ones. This knowledge can also constitute a base for later on researches when DAA became approved in pediatric age.

## 2. Methods

### 2.1. Study Population

This prospective cohort study included fifty children with CHC recruited from Pediatric Hepatology Department, National Liver Institute (a tertiary level institute), Menofiya University, Egypt, between June 2012 and June 2014. Their mean age was 11.46 ± 3.48 years and thirty three (66%) of them were males. Another 21 healthy children with comparable age (mean; 9.14 ± 3.31 years) and sex (males; 13 “61.9%”) to the disease group were enrolled as controls. CHC was diagnosed on the basis of positive anti-HCV antibodies and positive HCV-RNA for more than 6 months duration, together with the pathological picture of CHC [1]. Any case with associated liver disease (either identified by laboratory or histological examination) or out of the inclusion criteria for Peg/RBV therapy, as defined by El Naghi et al. [5], was excluded. A signed informed consent was obtained from parents of all recruited children before enrollment in the study. The study was approved by the Research Ethics Committee of the National Liver Institute, Menofiya University, and conforms to the 1975 Declaration of Helsinki and its later amendments.

### 2.2. Laboratory Investigations and Ultrasonographic Evaluation

Alanine transaminase (ALT), aspartate transaminase (AST), prothrombin time (PT), complete blood count (CBC), thyroid stimulating hormone (TSH), and serum autoantibodies (anti-nuclear antibodies, anti-smooth muscle antibodies, and liver-kidney microsomal antibodies) were performed for every patient.

Viral markers (anti-HCV, hepatitis B virus (HBV) surface antigen, HBV core immunoglobulin (Ig)M, and IgG antibodies) were performed for all patients using chemiluminescence immunoassay (Roche Diagnostic Inc., Mannheim, Germany). Real-time PCR for HCV-RNA was performed using Abbott m2000 real-time system (Abbott Molecular Inc., Des Plaines, Illinois, USA) for every patient before starting therapy and at week 12 of therapy. For those who continued therapy, PCR was tested at weeks 24 and 48 of treatment and lastly 6 months after the end of treatment. The detection limit was 15 IU/mL.

Abdominal ultrasound was performed using 2–5 MHz curved linear and 4–8 MHz linear transducers (Xario XG; Toshiba, Tokyo, Japan).

### 2.3. Liver Biopsy and Histopathological Evaluation

An ultrasonographic-guided liver biopsy was performed for all patients using a true-cut needle, size 16 G. Biopsy specimens were fixed in formalin, embedded in paraffin, and finally the slides obtained were stained by hematoxylin and eosin (H&E), perls, and orcein for routine histopathological evaluation.

Histological evaluation of chronic hepatitis was performed using Ishak et al. [14] scoring system. Grades of necroinflammatory activity 1–3 were ascribed as minimal, grades 4–7 as mild, and grades 8–12 as moderate, whereas grades >13 were ascribed as severe chronic hepatitis. Stages of fibrosis of 0-1 indicated absent/minimal fibrosis, stages 2-3 indicated significant fibrosis, and stages 4–6 indicated advanced fibrosis.

### 2.4. Peg/RBV Therapy

All children with CHC received Peg-IFN*α*-2b (PegIntron; Schering-Plough, New Jersey, USA) at a dosage of 60 *μ*g/m^2^/week subcutaneously and RBV orally at a dosage of 15 mg/kg/day on two divided doses. The duration of therapy for those who completed the course was 48 weeks. Virological responses of therapy were defined as reported by Ghany et al. [6]. Thirty-six (72%) children attained ETR and SVR with no relapses, while 14 (28%) children were nonresponders.

### 2.5. Serum Adiponectin, Vitamin D, and Alpha-Fetoprotein Assay

Serum samples were collected from every patient before starting Peg/RBV therapy and from healthy controls then stored at −80°C until used. Serum adiponectin level was determined by the Human Adiponectin (Acrp30) enzyme-linked immunosorbent assay (ELISA) kit (Orgenium Laboratories, Vantaa Finland). Serum vitamin D was measured using 25-OH Vitamin D Enzyme-Immunoassay (EIA) Kit (Immundiagnostik, Bensheim and Biomedica, Wien Austria) intended for the quantitative determination of the 25-OH vitamin D in plasma or serum. Lastly, serum AFP was measured by Quantikine, a human AFP EIA kit (R&D Systems Inc., Minneapolis, USA). Serum levels of the three test parameters were expressed as nanograms per milliliter (ng/mL).

### 2.6. Statistical Analysis

Values were expressed as mean ± standard deviation (range) or number (percentage) of individuals with a condition. For quantitative data, statistical significance was tested by either independent samples *t*-test or nonparametric Mann-Whitney *U* test according to the nature of the data. For qualitative data, significance was tested by Chi-square test or Fisher exact test. A multivariate analysis was performed using a binary logistic regression analysis for factors that significantly associated with treatment response on univariate analysis. Correlation was tested by Spearman test. The cutoff values for optimal clinical performance of adiponectin, vitamin D, AFP, and level of HCV-RNA for differentiation between responders and nonresponders were determined from the receiver-operating characteristic (ROC) curves. The diagnostic performance was presented as sensitivity, specificity, negative predictive value (NPV), positive predictive value (PPV), and accuracy. Results were considered significant if *p* value was <0.05. Statistical analysis was performed using SPSS, version 13 (SPSS Inc., Chicago, IL, USA).

## 3. Results

### 3.1. Baseline Demographic, Laboratory, and Histopathological Characteristics of the Studied Children

Body mass index of children with CHC was within average with a mean of 18.56 ± 2.79 kg/m^2^. Level of hepatitis C viremia ranged from 8.04 × 10^3^ to 6.23 × 10^6^ with a mean of 6.28 × 10^5^ ± 1.23 × 10^6^ IU/mL. Forty-five (90%) patients with CHC showed mild stage of fibrosis and 5 (10%) cases showed moderate fibrosis. Most of (86%) CHC children had mild activity, while 7 (14%) cases had minimal activity. Laboratory parameters are shown in Table 1.

### 3.2. Serum Adiponectin, Vitamin D and Alpha-Fetoprotein in Both CHC and Control Groups

**Figure 1:**
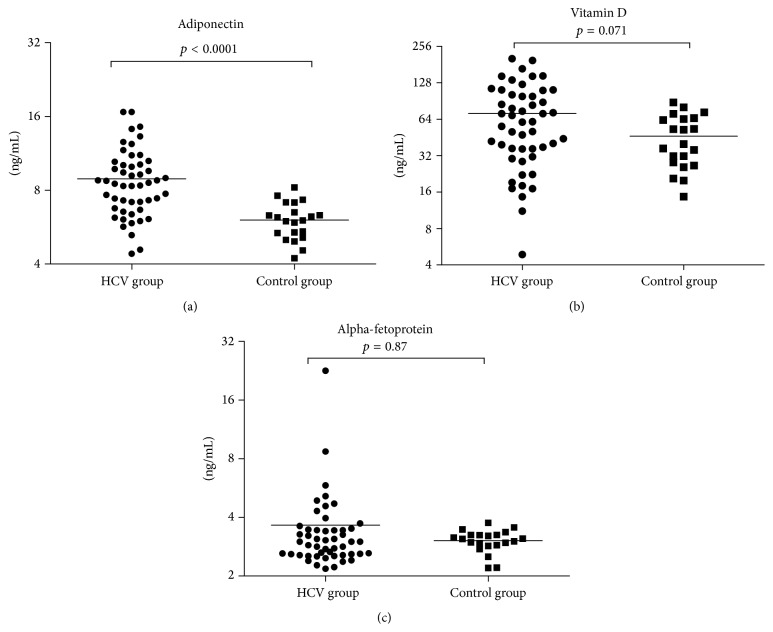
Comparison of pretreatment factors between HCV group and control group. (a) Adiponectin was significantly (*p* < 0.0001) higher in HCV group (8.92 ± 2.85 ng/mL, range; 4.4–16.7 ng/mL) than control group (6.049 ± 1.04 ng/mL, range; 4.2–8.2 ng/mL). (b) Vitamin D was insignificantly (*p* = 0.071) higher in HCV group (71.6 ± 49.1 ng/mL, range; 4.87–202 ng/mL) than control group (46.4 ± 21.8 ng/mL, range; 14.7–88 ng/mL). (c) AFP was insignificantly (*p* = 0.87) higher in HCV group (3.6 ± 2.96 ng/mL, range; 2.18–22.6 ng/mL) than control group (3.0 ± 0.39 ng/mL, range; 2.2–3.7 ng/mL).

Serum adiponectin was significantly higher in the CHC group than healthy controls (8.92 ± 2.85 and 6.049 ± 1.04 ng/mL, resp.; *p* < 0.0001). Also, in spite of being insignificant, serum vitamin D and AFP were higher in the CHC group (71.6 ± 49.1 ng/mL and 3.6 ± 2.96 ng/mL, resp.) than healthy controls (46.4 ± 21.8 ng/mL and 3.0 ± 0.39 ng/mL, resp.) (Figure 1).

### 3.3. Factors Associated with SVR

**Figure 2:**
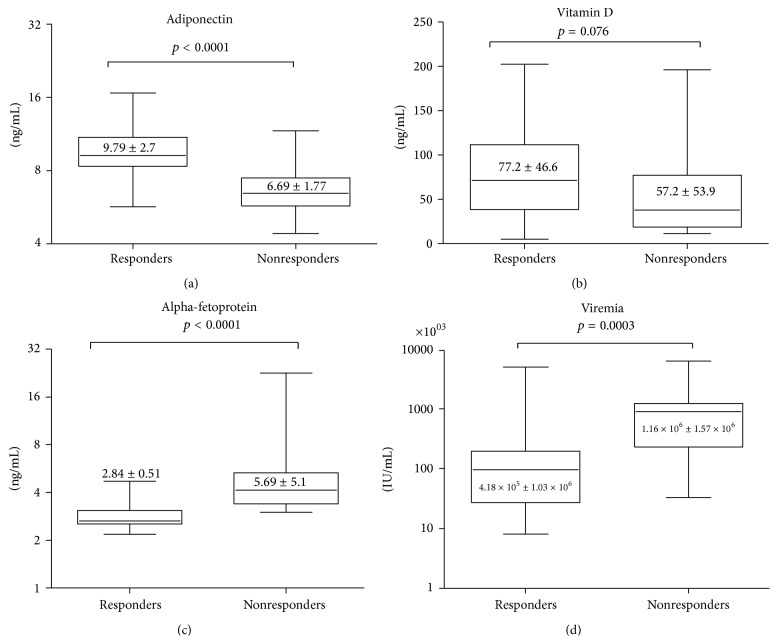
Comparison of pretreatment factors between responders and nonresponders. Box-and-whiskers plot for serum adiponectin, vitamin D, and AFP. The number indicated on the box represents mean ± standard deviation. (a) Adiponectin was significantly (*p* < 0.0001) higher in responders (9.79 ± 2.7 ng/mL, range; 5.68–16.68 ng/mL) than nonresponders (6.69 ± 1.77 ng/mL, range; 4.4–11.67 ng/mL). (b) Vitamin D was insignificantly (*p* = 0.076) higher in responders (77.2 ± 46.6 ng/mL, range; 4.9–202.4 ng/mL) than nonresponders (57.2 ± 53.9 ng/mL, range; 11.1–196 ng/mL). (c) AFP was significantly (*p* < 0.0001) lower in responders (2.84 ± 0.51 ng/mL, range; 2.18–4.7 ng/mL) than nonresponders (5.69 ± 5.1 ng/mL, range; 3–22.6 ng/mL). (d) Viremia was significantly (*p* = 0.0003) lower in responders (4.18 × 10^5^ ± 1.03 × 10^6^ IU/mL, range; 8.04 × 10^3^–4.96 × 10^6^ IU/mL) than nonresponders (1.16 × 10^6^ ± 1.57 × 10^6^ IU/mL, range; 3.29 × 10^4^–6.24 × 10^6^ IU/mL).

Pretreatment factors that could be associated with response to peg/RBV therapy were compared between treatment responders (SVR) and treatment nonresponders (Figure 2). It was found that adiponectin was significantly higher in those with SVR (9.79 ± 2.7 versus 6.69 ± 1.77 ng/mL; *p* < 0.0001). On the other hand it was found that both AFP and viremia were significantly lower in the treatment responders (2.84 ± 0.51 ng/mL and 4.18 × 10^5^ ± 1.03 × 10^6^ IU/mL, resp.) than in nonresponders (5.69 ± 5.1 ng/mL and 1.16 × 10^6^ ± 1.57 × 10^6^ IU/mL, resp.) with *p* < 0.0001 and *p* = 0.0003, respectively. Lastly, vitamin D was found to be higher in the treatment responders (77.2 ± 46.6 ng/mL) than nonresponders (57.2 ± 53.9 ng/mL), with borderline significance, *p* = 0.076. Other studied pretreatment parameters showed no difference between responders and nonresponders (Table 1), while in multivariate analysis adiponectin was shown to be the only significant independent predictor of treatment response (*p* = 0.044) (Table 3).

### 3.4. Diagnostic Performance of Predictors of Treatment Response

**Figure 3:**
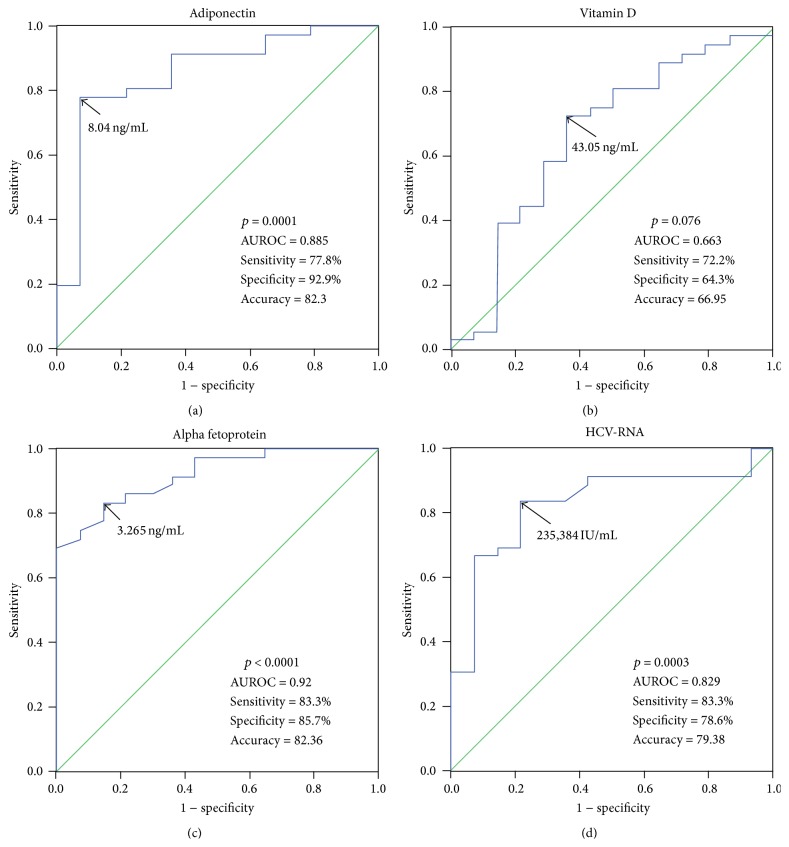
Diagnostic performance of pretreatment parameters for differentiation between responders and nonresponders. Receiver-operating characteristic (ROC) curves of (a) adiponectin, (b) vitamin D, (c) AFP, and (d) HCV-RNA level for differentiation between responders and nonresponders. The arrows indicate the cutoff values. AUROC: area under ROC.

Cutoff points for variables showing significant associations with treatment response were analyzed by the ROC curves (Figure 3). For adiponectin it was found that at a cutoff value of >8.04 ng/mL, it can predict treatment response by 77.8% sensitivity, 92.9% specificity, 96.6% PPV, 61.9% NPV, and 82.3% accuracy, while AFP and HCV-RNA at cutoff values <3.265 ng/mL and <235,384 IU/mL, respectively, can predict treatment response with a sensitivity of 83.3% and 83.3%, specificity of 85.7% and 78.6%, PPV of 93.75% and 90.9%, NPV of 66.7% and 64.7%, and accuracy of 82.36% and 79.38%, respectively.

### 3.5. Correlation of Predictors of Treatment Response with Other Studied Parameters

Adiponectin was found to be significantly negatively correlated with both AFP (*r* = −0.29 and *p* = 0.043) and level of viremia (*r* = −0.39 and *p* = 0.005), with no significant correlation with other studied parameters. Also, there was no significant correlation between AFP, vitamin D, and level of viremia and all other studied parameters (Table 2).

## 4. Discussion

This study is, to our knowledge, the first to show the predictive value of baseline serum levels of adiponectin, vitamin D, and AFP for the treatment response of CHC in children.

Adiponectin is an adipocytokine secreted by adipocytes. It is a protein hormone that modulates a number of metabolic processes including glucose and fatty acid catabolism. Also it has been suggested that adiponectin has a hepatoprotective role [15]. The anti-inflammatory effects of adiponectin could protect the liver from the development of inflammation and cell injury [16].

In the present work, the significantly higher adiponectin in the CHC group than the control group may be due to an anti-inflammatory role of adiponectin in those with CHC. In previous studies, adiponectin was found to directly affect the inflammatory response by regulating both production and activity of cytokines [16]. In addition, hypoadiponectinemia has been reported to enhance hepatic steatosis, inflammation, fibrosis, and hepatocarcinogenesis in animal models of liver diseases [17–19]. Moreover, nonalcoholic steatohepatitis patients show lower levels of adiponectin with higher grades of inflammation [15].

In this work, the pretreatment serum level of adiponectin was significantly higher in the treatment responders (SVR) than nonresponders, and at a cutoff value of >8.04 ng/mL it can predict the treatment response by a sensitivity of 77.8% and a specificity of 92.9%. Zografos et al. [20] found that lower adiponectin was an independent predictor of no virological response at the end of treatment (*p* < 0.001). This may indicate the benefit of the anti-inflammatory role of adiponectin [16] in those with CHC. Adiponectin administration, in both alcoholic and nonalcoholic fatty liver in mice, was found to suppress hepatic production and the circulating levels of tumor necrosis factor-*α* and ameliorates hepatic steatosis [21].

Moreover, the significant negative correlation found in this work between serum adiponectin and viremia may indicate an antiviral role of adiponectin. On the other hand, it may suggest that HCV may directly affect adiponectin. This later concept was suggested by the study of Zografos et al. [20] who found a significant increase of adiponectin at the end of HCV treatment for those with ETR.

Abdel Latif et al. [22] found that serum adiponectin levels were lower in HCV-infected patients with steatosis than in those without steatosis and these levels tend to decrease with the increase in the grade of steatosis, the advance in the grade of histological activity, and the stage of fibrosis. In our work, there was no significant correlation between adiponectin and stage of fibrosis or grade of necroinflammatory activity. This difference may relate to the difference in age range and the relatively mild histological affection of our CHC group.

According to the previous results, not only can adiponectin be used as a reliable pretreatment predictor of treatment response in combination with other defined parameters but also it can be tried as an adjuvant therapy with peg/RBV especially for those with pretreatment lower serum levels. To prove this, it needs a well controlled clinical trial.

Beside its action in calcium homeostasis, vitamin D has a significant immunomodulatory action and is an important mediator of innate and adaptive immune systems [23]. In spite of many researches, no strict data are found on the relationship between vitamin D and CHC. Generally, in relation to vitamin D synthesis in the liver, mild to moderate liver dysfunction causes malabsorption of vitamin D. Moreover, liver dysfunction of 90% or more results in inability to make sufficient 25-OH vitamin D [24]. Some researchers showed that adults with CHC have higher incidence of severe 25-OH vitamin D deficiency compared to the normal control [25]. On the contrary, in the present study we found that vitamin D was higher in children with CHC than normal controls with borderline significance (*p* = 0.071). This difference from Lange et al. [25] study may be due to younger age and milder liver affection in our children compared to their adult population. Hypothetically, this reported vitamin D increase in children with CHC reflects a possible antiviral role of vitamin D. This is supported by Matsumura et al. [26] who demonstrated in in vitro study that 25-OH vitamin D is an anti-HCV agent that targets viral particle assembly step.

Petta et al. [27] found that low vitamin D serum level is related to severe fibrosis in adults with CHC. However, we did not find significant correlation between vitamin D and any of the stage of fibrosis or grade of activity or any other studied pretreatment parameter.

Vitamin D concentration has emerged recently as a new predictor of HCV treatment response. This novel predictor is of great interest because it is easily modifiable by supplementation [28]. However, no previous data are available for the pediatric age group.

In the present work, a higher vitamin D level was found in HCV treatment responders than nonresponders but with borderline significance. Similar to our results Petta et al. [27], Bitetto et al. [28], and Nimer and Mouch [29] detected an association between lower vitamin D serum levels and failure to achieve SVR in adults with CHC. On the other hand, Lange et al. [25] found that pretreatment serum level of vitamin D is not an optimal predictor of treatment response in HCV genotype 1.

Hypothetically, in the presence of vitamin D deficiency, it might be preferable to correct the deficiency before starting antiviral therapy. Nevertheless, to date there are few published reports on the role of vitamin D supplementation in patients with CHC. Bitetto et al. [30] found that vitamin D supplementation, in adults with recurrent hepatitis C postliver transplant, improves the probability of achieving a SVR. Also, Nimer and Mouch [29] found that adding vitamin D to conventional Peg/RBV therapy for patients with HCV genotypes 2-3 significantly improved viral response.

The reported higher vitamin D among treatment responders in the present study together with the known immunomodulatory action of vitamin D [23] and previous clinical trials of vitamin D supplementation in adults [29, 30] suggest that adding vitamin D to Peg/RBV therapy in children may increase SVR rates without serious adverse events. However, to prove these findings, well designed and large prospective studies are needed.

Serum AFP is a fetal glycoprotein produced by the yolk sac and fetal liver. Following birth, AFP levels decrease rapidly to less than 20 ng/mL and increase significantly in certain pathologic conditions. Serum AFP is a routinely used marker for hepatocellular carcinoma [31]. Yet, significant elevations of AFP are commonly seen in nonhepatic malignancies and benign conditions, such as acute and chronic viral hepatitis [32]. Previous studies reported that the prevalence of increased serum AFP varies from 10% to 43% in adult patients with CHC and suggested an association between an increased serum AFP and advanced fibrosis or cirrhosis [33–36].

In agreement with the above reports, we found that AFP was higher in children with CHC than healthy controls with statistically insignificant difference. The absence of a significant correlation between AFP and stage of fibrosis in the present work may be due to the milder liver affection with low stages of fibrosis in reported cases when compared to the adult series.

Some studies found significant correlation of pretreatment low AFP serum level and treatment response in adults with CHC [37–39]. In accordance with the previous studies, we found that lower serum AFP was significantly related to SVR in children with CHC but with borderline significance in multivariate analysis. The SVR was 93.75% among children with AFP below 3.265 ng/mL and 33.3% for children with AFP ≥ 3.265 ng/mL.

The link between AFP and treatment response needs further studies. Increased production of AFP in hepatitis and cirrhosis was first thought to reflect the process of surviving hepatocytes, but this hypothesis has been refuted by other reports [33, 40]. More recent hypothesis ascribes the increased serum AFP to the hepatic damage* per se* with selective transcriptional activation of the AFP gene [32]. In the present study, the reported negative correlation between adiponectin with its known hepatoprotective role [15] and AFP (*p* = 0.043) is supporting this hypothesis and may give an explanation to the link between the baseline serum AFP and the treatment response.

In the present study viral load in univariate analysis was found to be a significant pretreatment predictor of treatment response as it was shown in previous reports [6]. However, in multivariate analysis, it was not an independent predictor of treatment response.

The emergence of the new DAAs for the treatment of CHC [41, 42] may be considered a limitation in the current study. However, peg/RBV is the still approved standard therapy for children with CHC [5, 43]. Moreover, Peg-IFN and RBV are still included with DAAs in the treatment regimens of those with genotype 4 [41], the prevalent genotype in Egypt [7]. A major limitation of this study is the low number of cases and controls enrolled. Also, absence of testing for HCV genotype and* IL28B* genotype could be considered another limitation of this work.

## 5. Conclusions

In conclusion, serum adiponectin can be added to the list of the pretreatment determinants of SVR in children with CHC, with the advantage of being easy to perform, noninvasive, and modifiable. In spite of its significant prediction for the likelihood of response, we cannot state to screen and select the patients to be treated or not based on their pretreatment levels of this predictor. Instead, it can be used to prioritize patients to treatment when resources are limited, thus avoiding toxicities and cost for those unlikely to respond to treatment. Also as it is a modifiable factor, supplementation for deficient cases could be considered before starting treatment if this could be proved in further studies.

## Figures and Tables

**Table 1:** Baseline demographic, laboratory, and histopathological characteristics of the hepatitis C virus-infected group.

Item	All (*n* = 50)	Responders (*n* = 36)	Nonresponders (*n* = 14)	*p* value
Age (years)	11.46 ± 3.48 (4–18)	11.8 ± 3.6 (4–18)	10.6 ± 3.1 (5–16)	0.237
Sex (male)	33 (66%)	25 (69.4%)	8 (57.1%)	0.41
BMI	18.56 ± 2.79 (14.21–25.36)	18.8 ± 2.9 (14.2–25.4)	17.98 ± 2.4 (14.8–21.1)	0.334
Hemoglobin (g/dL)	11.7 ± 0.76 (10–13.2)	11.6 ± 0.75 (10–13.1)	11.9 ± 0.75 (10.3–13.2)	0.209
White blood cells (×10^3^/*μ*L)	7.3 ± 1.8 (4.5–12.3)	7.3 ± 1.6 (4.5–11.8)	7.3 ± 2.2 (4.8–12.3)	0.892
Neutrophils (×10^3^/*μ*L)	5.14 ± 1.2 (3.24–8.14)	5.1 ± 1.13 (3.24–8.14)	5.2 ± 1.4 (3.3–7.6)	0.828
Platelets (×10^3^/*μ*L)	242 ± 82 (122–431)	238 ± 84 (122–431)	253 ± 76 (123–351)	0.544
ALT (U/L)	56.4 ± 13.6 (40–108)	56.3 ± 12.3 (40–86)	56.6 ± 17.1 (40–108)	0.943
AST (U/L)	62.9 ± 25.7 (35–157)	63 ± 28.1 (35–157)	61.9 ± 19.4 (40–110)	0.665
Stage of fibrosis				0.23
Absent/minimal	29 (58%)	19 (52.8%)	10 (71.4%)	
Significant	21 (42%)	17 (47.2%)	4 (28.6%)	
Grade of activity				0.345
Minimal	7 (14%)	4 (11.1%)	3 (21.4%)	
Mild	43 (86%)	32 (88.9%)	11 (78.6%)	

HCV: hepatitis C virus; BMI: body mass index; ALT: alanine transaminase, AST: aspartate transaminase. *p* value is for the comparison between responders and nonresponders.

**Table 2:** Correlation of predictors of treatment response with other studied parameters.

Item	Adiponectin		Alpha-fetoprotein		HCV-RNA		Vitamin D	
	*r*	*p*	*r*	*p*	*r*	*p*	*r*	*p*
Alpha-fetoprotein	−0.29	**0.043**						
HCV-RNA	−0.39	**0.005**	0.217	0.13				
Vitamin D	0.124	0.391	−0.09	0.545	−0.06	0.66		
Age	0.055	0.705	0.05	0.731	0.042	0.773	0.203	0.157
BMI	0.045	0.755	0.223	0.119	−0.03	0.812	0.129	0.372
HB	−0.04	0.765	0.04	0.782	−0.02	0.882	0.028	0.85
WBCs	0.096	0.508	−0.14	0.315	0.032	0.823	0.001	0.996
Neutrophils	0.123	0.396	−0.10	0.474	0.067	0.644	−0.01	0.953
Platelets	−0.21	0.142	−0.04	0.795	0.127	0.379	0.027	0.855
ALT	0.06	0.677	−0.06	0.705	−0.06	0.666	−0.04	0.768
AST	0.162	0.262	0.077	0.594	0.153	0.287	−0.06	0.677
Stage of fibrosis	0.189	0.189	−0.09	0.524	0.016	0.911	0.141	0.327
Grade of activity	0.084	0.563	0.166	0.25	0.049	0.737	0.073	0.616

HCV-RNA: hepatitis C virus ribonucleic acid; BMI: body mass index; HB: hemoglobin; WBCs: white blood cells; ALT: alanine transaminase; AST: aspartate transaminase.

**Table 3:** Multiple regression analysis for pretreatment predictors of treatment response.

Item	*β*	*p* value	95% CI	
			Lower	Upper
HCV-RNA by PCR (IU/mL)	−0.000002	0.119	1.0	1.0
Alpha-fetoprotein (ng/mL)	−8.51	0.056	0.00000003	1.24
Adiponectin (ng/mL)	3.142	0.044	1.095	489.93

Β: regression coefficient; CI: confidence interval.
